# The Impacts of Aspergillosis on Outcome, Burden and Risks for Mortality in Influenza Patients with Critical Illness

**DOI:** 10.3390/jof7110922

**Published:** 2021-10-29

**Authors:** Chien-Ming Chao, Chih-Cheng Lai, Hsuan-Fu Ou, Chung-Han Ho, Khee-Siang Chan, Chun-Chieh Yang, Chin-Ming Chen, Wen-Liang Yu

**Affiliations:** 1 Chi Mei Medical Center, Department of Intensive Care Medicine, Liouying, Tainan 73657, Taiwan; ccm870958@yahoo.com.tw; 2 Department of Dental Laboratory Technology, Min-Hwei College of Health Care Management, Tainan 73657, Taiwan; 3 Department of Internal Medicine, Kaohsiung Veterans General Hospital, Tainan Branch, Tainan 71051, Taiwan; dtmed141@gmail.com; 4 Chi Mei Medical Center, Department of Intensive Care Medicine, Chiali, Tainan 72263, Taiwan; Iamkiu@gmail.com; 5 Chi Mei Medical Center, Department of Medical Research, Tainan 71004, Taiwan; ho.c.hank@gmail.com; 6 Department of Hospital and Health Care Administration, Chia Nan University of Pharmacy & Science, Tainan 71710, Taiwan; 7 Chi Mei Medical Center, Department of Intensive Care Medicine, Yongkang, Tainan 71004, Taiwan; kheesiangchan@gmail.com (K.-S.C.); ycc851205@gmail.com (C.-C.Y.); chencm3383@gmail.com (C.-M.C.); 8 Department of Medicine, School of Medicine, College of Medicine, Taipei Medical University, Taipei 11031, Taiwan

**Keywords:** aspergillosis, influenza, outcome, intensive care unit, mortality

## Abstract

Previous studies have revealed higher mortality rates in patients with severe influenza who are coinfected with invasive pulmonary aspergillosis (IPA) than in those without IPA coinfection; nonetheless, the clinical impact of IPA on economic burden and risk factors for mortality in critically ill influenza patients remains undefined. The study was retrospectively conducted in three institutes. From 2016 through 2018, all adult patients with severe influenza admitted to an intensive care unit (ICU) were identified. All patients were classified as group 1, patients with concomitant severe influenza and IPA; group 2, severe influenza patients without IPA; and group 3, severe influenza patients without testing for IPA. Overall, there were 201 patients enrolled, including group 1 (*n* = 40), group 2 (*n* = 50), and group 3 (*n* = 111). Group 1 patients had a significantly higher mortality rate (20/40, 50%) than that of group 2 (6/50, 12%) and group 3 (18/11, 16.2%), *p* < 0.001. The risk factors for IPA occurrence were solid cancer and prolonged corticosteroid use in ICU of >5 days. Group 1 patients had significantly longer hospital stay and higher medical expenditure than the other two groups. The risk factors for mortality in group 1 patients included patients’ Charlson comorbidity index, presenting APACHE II score, and complication of severe acute respiratory distress syndrome. Overall, IPA has a significant adverse impact on the outcome and economic burden of severe influenza patients, who should be promptly managed based on risk host factors for IPA occurrence and mortality risk factors for coinfection with both diseases.

## 1. Introduction

Invasive pulmonary aspergillosis (IPA) could be associated with high morbidity and mortality [1,2,3]. Although IPA typically occurs in immunocompromised patients, more and more IPA cases reportedly develop in non-classically immunocompromised hosts [4,5,6]. The risk factors of IPA in critically ill patients without immunocompromised conditions include the use of corticosteroids, chronic obstructive pulmonary disease, acute respiratory distress syndrome, hepatic failure, and multiple organ dysfunction [4,5]; moreover, influenza itself is an independent risk factor of IPA and is associated with high mortality [7,8,9,10,11,12,13]. IPA might affect up to 23–29% of severe influenza patients [14,15,16]. However, the impacts of IPA on the outcome of critically ill influenza patients remain variable around the world, probably due to inconsistent definitions and diagnostic criteria of IPA during clinical practice for managing influenza patients among different institutes. In Taiwan, it has not been confirmed whether IPA increases the mortality of the patients with severe influenza. In addition, the host factors contributing to IPA in critically ill influenza patients and their mortality risk factors remain unidentified. Therefore, we conducted a 3-year multicenter study to delineate the impacts of IPA on the clinical outcome and economic burden among influenza patients staying in ICUs. We also aimed to identify the risk factors for IPA occurrence as well as the risk factors for mortality of IPA patients who also have severe influenza.

## 2. Methods

### 2.1. Study Design

This study was conducted in three hospitals, including one medical center, one regional hospital, and one district hospital in southern Taiwan based on a retrospective study. From 2016 to 2018, all adult patients with severe influenza admitted to an ICU in either of these three hospitals were identified, and only the first time of ICU admission was included. Among them, the diagnosis of IPA was surveyed. All studied patients were classified into the following three groups: (1) patients with concomitant severe influenza and IPA (designed group 1: Flu with IPA); (2) severe influenza patients without IPA (designed group 2: Flu without IPA); and (3) severe influenza patients without galactomannan (GM) testing for IPA (designed group 3: Flu without GM test). Their clinical data included gender, age, underlying disease/conditions, recent use of corticosteroid, types and subtypes of influenza, radiographic findings on the day of arranging IPA testing, disease severity using Acute Physiology and Chronic Health Evaluation II (APACHE II) score at ICU admission, sequential organ failure assessment (SOFA) scores, antifungal treatment, and organ supports. The primary outcome was all-cause mortality in the ICU. Laboratory data were obtained from the electronic resources of the Chi Mei medical systems.

### 2.2. Definition

As a previous study [9] described, influenza was confirmed if the patients had one positive result of the following tests including rapid influenza diagnostic tests (RIDTs); real-time polymerase chain reaction (PCR) for influenza A, influenza B, influenza A (H1N1), and influenza A (H3N2); and viral isolation for specimens of nasopharyngeal swab and/or lower respiratory tract aspirates. Influenza was defined as severe in those influenza patients requiring ICU admission.

Positive *Aspergillus* GM antigen was defined as the value of an optical density index ≥0.5 in serum and/or ≥0.7 in fluid from bronchoalveolar lavage (BAL) using s Platelia *Aspergillus* Ag assay (Bio-Rad Laboratories, Marnes-La-Coquette, France) [17].

In China, Zhou et al. reported that the sensitivity and specificity of BAL GM detection at a cutoff value of ≥1.0 was 64.86% and 90.36%, respectively. However, receiver operating characteristic curve analysis showed that the optimized diagnostic cutoff value of BAL GM for pulmonary aspergillosis was 0.7, and the sensitivity and specificity reached 72.97% and 89.16%, respectively [17]. BAL GM detection was valuable for the diagnosis of IPA in nonneutropenic patients; therefore, we adapted 0.7 as the optimal BAL GM cutoff value for IPA in severe influenza patients in Taiwan.

In this study, proven IPA was defined as the presence of histopathologic evidence on a specimen obtained by lung biopsy, in which branching hyphae are seen accompanied by evidence of associated tissue damage [18]. A probable IPA diagnosis was considered as a patient with (1) a host factor at least of severe influenza, but not necessarily limited to a classically immunocompromised status [14,15,16]; (2) clinical features of infections signs, worsening respiratory insufficiency in spite of appropriate management, and medical imaging based on the presence of a halo sign, air crescent sign, cavity, wedge-shaped, and segmental or lobar consolidation, or acute pulmonary infiltrates of the lungs; and (3) mycological evidence with positive GM antigen in the serum and/or BAL fluid [10,19]. The proven or probable IPA was enrolled in group 1 patients. We did not define “possible IPA” for influenza patients, as those with negative GM testing or not testing GM assay were enrolled into group 2 and group 3 patients, respectively. In this study, the order of GM assays from clinical specimens including blood were requested by treating physicians. Coinfection was defined by the identification of other respiratory pathogens within 2 days of diagnosing influenza infection [20].

### 2.3. Statistical Analysis

The clinical characteristics among groups were compared using the χ2 test or Fisher exact test for categorical variables. ANOVA or Welch’s ANOVA test was used for continuous variables if appropriate, while Kaplan–Meier estimates were used to display time-to-survival rates by group. The odds ratio analysis was used to estimate the relative risks for IPA occurrence or hospital mortality using logistic regression model. Firstly, the above-analyzed significant baseline characteristics, including underlying disease, comorbidity, disease severity, and complications, and other potentially confounding variables were screened by the univariate analysis. Secondly, all variables with *p*-values < 0.05 from the univariate analysis were included in the multivariable regression model. A two-tailed *p*-value < 0.05 was considered statistically significant. All statistics were performed using IBM SPSS Statistics version 18.

## 3. Results

### 3.1. Study Subjects

**Figure 1:**
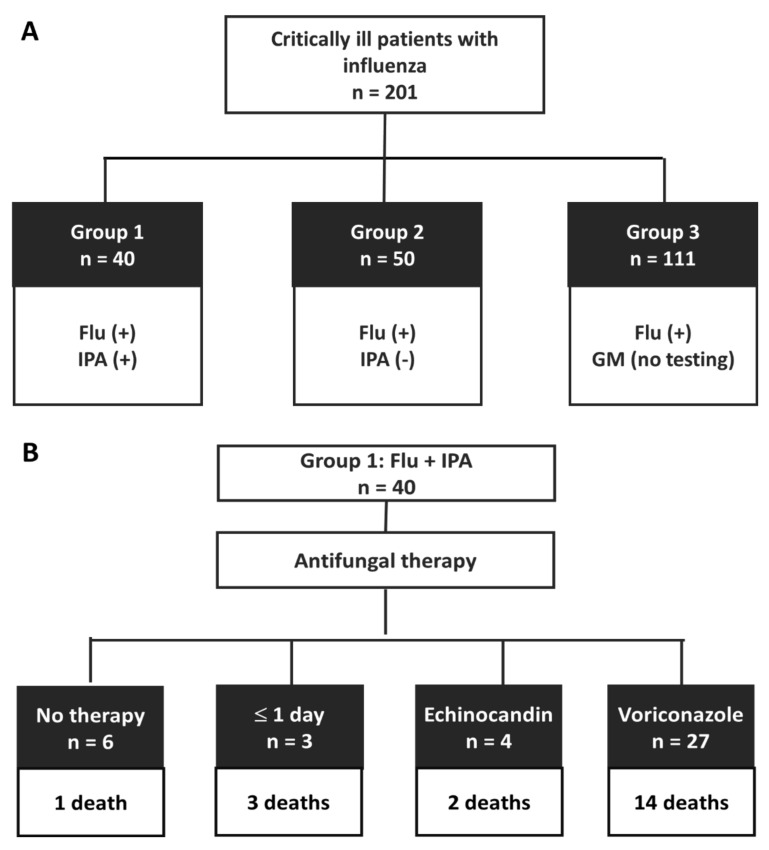
A total of 201 critically ill patients with positive testing for influenza were enrolled and were classified into three groups (**A**) and clinical outcome of group 1 (**B**) in real-world data from 2016 to 2018. Note. Flu, influenza assay; GM, galactomannan assay; (+), positive result; (-), negative result.

During the 3-year period from 2016 to 2018, a total of 201 critically ill patients with positive tests for influenza were included (Figure 1A). Among them, 40 (19.9%) patients had concomitant IPA (group 1), 50 patients tested negative for IPA (group 2), and 111 patients did not receive any test for IPA (group 3). Among group 1 patients, the days of influenza diagnosis after hospitalization ranged from −8 to 24 days, with a mean value of 2.1 (standard deviation, 5.0) days. Two patients had influenza diagnosis before admission (−8 and −3 days); 18 patients had influenza diagnosis on the same day of admission; 18 patients were diagnosed influenza within 2 weeks subsequently; and two patients had influenza diagnosis after 2 weeks of hospitalization (15 and 24 days). The days of confirming IPA after influenza diagnosis ranged from −1 to 64 days, with a mean value of 7.5 (standard deviation, 10.8) days. One patient had IPA diagnosis before diagnosing influenza (−1 day); 36 patients were diagnosed IPA within 2 weeks post influenza diagnosis; and three patients had IPA diagnosis after 2 weeks of diagnosing influenza (15, 33, and 64 days respectively).

The clinical specimens of GM testing positive in group 1 (*n* = 40) included BAL (*n* = 5), blood sample (*n* = 36), and both BAL and blood sample (*n* = 1). The positive GM data in BAL were 0.78, 2.39, 7.49, 8.19, and 9.02 index in five patients, respectively. The positive GM data in serum ranged from 0.5 to 13.63 index, with a mean value of 1.77 (standard deviation, 2.42) index. Three patients had positive *Aspergillus* growth from the sputum. The category of IPA for all the patients in group 1 was probable IPA, as no biopsy specimens were obtained. The specimens of GM testing negative in group 2 (*n* = 50) included BAL (*n* = 3), blood sample (*n* = 50), and both BAL and blood sample (*n* = 3). The negative GM data in BAL were 0.07, 0.09, and 0.44 index in three patients, respectively. The negative GM data in serum ranged from 0.05 to 0.39 index, with a mean value of 0.17 (standard deviation, 0.10) index.

### 3.2. Clinical Characteristics of Patients with Severe Influenza

Among 201 patients with severe influenza, influenza A (*n* = 181) was the most common virus type followed by influenza B (*n* = 20). H3N2 (*n* = 93) was the most common subtype of influenza A followed by H1N1 (*n* = 60), and non-H1N1 and non-H3N2 (*n* = 28). Group 1 (*n* = 40) included 33 patients with influenza A (15 H1N1, 3 H3N2, and 15 others), and 7 patients with influenza B. Group 1 patients had a lower portion of influenza A (H3N2) than the other two groups. In contrast, group 1 patients had higher portion of negative RIDT or influenza A (non-H1N1 and non-H3N2) than other groups (Table 1).

Significant differences in the distribution of hospitals, age, the frequency of underlying solid cancer, the use of corticosteroids, SOFA scores, CMV DNAemia, the presence and severe severity of ARDS, and lymphopenia were significantly higher in group 1 than in the other two groups except for younger age (Table 1). Group 1 had significantly higher proportion of steroid use in ICUs (*p* = 0.001), while group 3 had significantly less steroid use (total dose and duration) as well as ventilator use than the other two groups; moreover, group 1 had the worst outcomes, including prolonged ICU stay, length of hospital stay, and all-cause mortality rate, and was associated with highest economic burden of hospital cost (all *p* < 0.001). The above-mentioned significant variables in group 1 almost reached statistically significant difference in comparison to those of group 2, except for hospital distribution, total dosage, and duration of corticosteroid use (Table 1).

**Figure 2:**
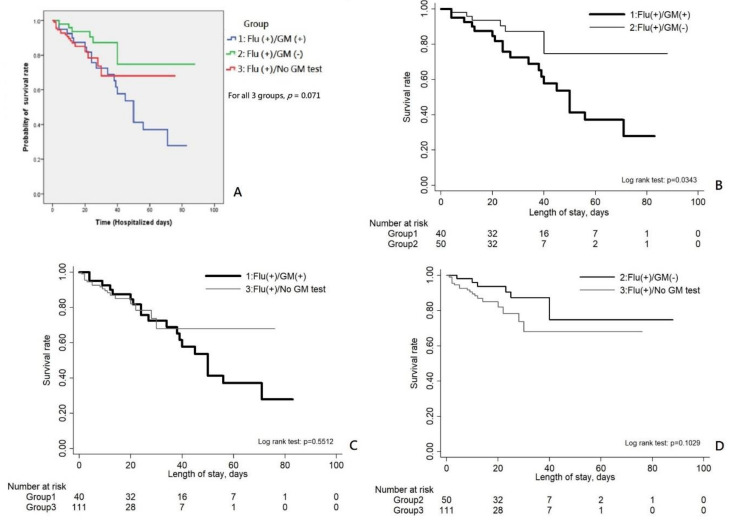
The overall survival using time-to-event analysis was lowest for group 1 of severely ill influenza patients with concomitant IPA among all three influenza groups (*p* = 0.071, (**A**)) with significant difference to group 2 with severe influenza only (*p* = 0.034, (**B**)). There was no significant difference between group 1 and group 3 (*p* = 0.551, (**C**)) and between group 2 and group 3 (*p* = 0.103, (**D**)). Note. Flu, influenza assay; GM, galactomannan assay; (+), positive result; (-), negative result.

The overall survival using time-to-event analysis was lowest for group 1 of severe influenza patients with concomitant IPA among all three influenza groups (*p* = 0.071, Figure 2). For further comparison, the time-to-survival event of group 1 was significantly lower than that of group 2 (*p* = 0.034), but was not significantly different to that of group 3 (*p* = 0.551), while time-to-survival event of group 2 was not significantly different to that of group 3 (*p* = 0.103).

### 3.3. Clinical Characteristics of Patients with Coinfection of IPA and Influenza

Among 40 patients with co-IPA and influenza infection, 31 (77.5%) were diagnosed in the medical center and males comprised 60% of patients. Diabetes mellitus was the most common underlying disease (*n* = 19, 47.5%), followed by solid cancer (*n* = 9, 22.5%) including urological cancer (*n* = 4), head and neck cancer (*n* = 2), gastric cancer (*n* = 2), and cervical cancer (*n* = 1).

In comparison to group 2, the significant risk factors for development of IPA in influenza patients (group 1) included solid cancer (odds ratio, 6.97, 95% CI, 1.41–34.4, *p* = 0.017), prolonged steroid use in ICU of >5 days (odds ratio, 4.38, 95% CI, 1.73–11.10, *p* = 0.002), and steroid total dose (odds ratio, 1.001, 95% CI, 1.001–1.002, *p* = 0.041) using univariate logistic regression, but only solid cancer (*p* = 0.015) and prolonged steroid use in ICU (*p* = 0.002) reached statistical significance using multivariate analysis. Patients with influenza A (H3N2) infection were less likely to develop IPA (odds ratio, 0.13, 95% CI, 0.036–0.489, *p* = 0.002). Other potential risk factors for IPA occurrence did not reach statistical significance, including daily steroid dose (odds ratio, 1.015, 95% CI, 0.988–1.043, *p* = 0.282), CMV DNAemia (odds ratio, 2.97, 95% CI, 0.81–10.9, *p* = 0.101), ARDS (odds ratio, 2.65, 95% CI, 0.98–7.20, *p* = 0.056), severe degree of ARDS (odds ratio, 1.95, 95% CI, 0.73–5.25, *p* = 0.085), negative RIDT (odds ratio, 1.91, 95% CI, 0.75–4.85, *p* = 0.174), Influenza A (H1N1) infection (odds ratio, 0.98, 95% CI, 0.42–2.31, *p* = 0.961), and lymphopenia (odds ratio, 2.43, 95% CI, 0.89–6.63, *p* = 0.083).

Twenty-five (62.5%) patients required mechanical ventilation support and one (2.5%) needed extracorporeal membrane oxygenation. Septic shock was the most common complication (*n* = 39, 97.5%), followed by acute kidney injury (*n* = 15, 37.5%). The chest radiograph presented was based on the features on the day of initiating GM testing. Bilateral extensive consolidation and multiple patches with necrotizing processes were found in one-fourth of the patients. Bacterial coinfection was found in 18 (45%) patients, while influenza A (H1N1) was the most common type of influenza infection. Twenty (50%) patients required prolonged ICU stay (>21 days) and the overall all-cause mortality rate was 50%.

### 3.4. Outcome of IPA-Coinfected Influenza Patients Receiving Antifungal Therapy

Firstly, nine patients in group 1 received inadequate antifungal therapy for less than 1 day (*n* = 3) or did not receive any antifungal therapy (*n* = 6). The clinical characteristics of the nine patients are tabulated in Table 2. Among the six patients without antifungal therapy, four patients did not require respiratory ventilator support; four patients had a relative low serum GM level (0.5–0.78 index); and all patients did not have a severe degree of ARDS. Only one (16.7%) patient died among those who did not receive any antifungal treatment.

**Figure 3:**
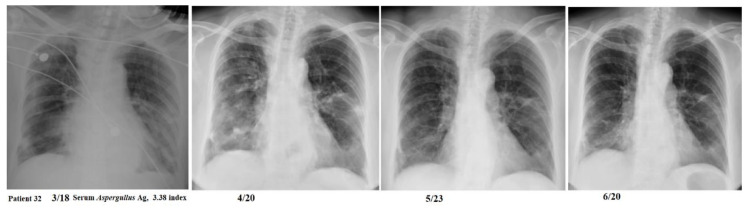
A 64-year-old woman had a serum *Aspergillus* galactomannan test of 3.38 index and did not receive any antifungal therapy but experienced a prolonged course of more than 3 months for resolution of the bilateral pulmonary infiltrations.

**Figure 4:**
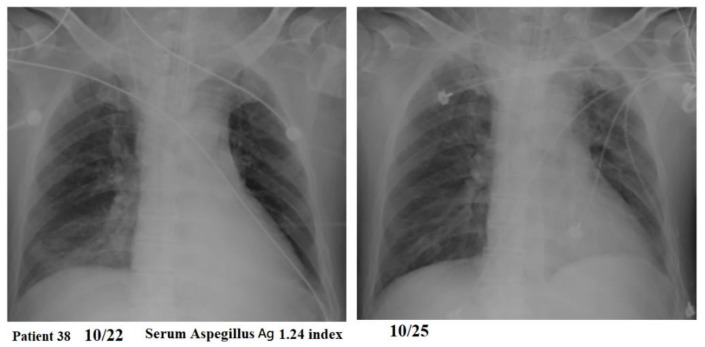
A 48-year-old man had a serum *Aspergillus* galactomannan test of 1.24 index and did not receive any antifungal therapy but experienced a rapid course of resolution of the pulmonary infiltrates over the right lower lung field.

Patients might experience a rapid or prolonged course of resolution of the pulmonary infiltrates (Figure 3 and Figure 4). Among the three patients who could not receive adequate antifungal therapy in time, one patient (patient 3) rapidly died within 4 days of hospital stay and IPA was diagnosed 3 days after discharge (7 days after influenza diagnosis). One patient (patient 25) had a late diagnosis of IPA on the day before death during a prolonged course of hospital stay for 71 days. One patient (patient 30) had an early diagnosis of IPA and rapidly died within 4 days of hospital stay.

Secondly, the overall mortality rate in group 1 was 20/40 (50%). Among the 31 patients with more than three days of antifungal therapy regarded as a treatment group (Figure 1B), four patients received an echinocandin as alternative therapy (Table 1). These four patients had at least a potential risk for voriconazole intolerance, such as liver function impairment, cirrhosis, jaundice, or prolonged QTc interval on electrocardiogram. Two patients died in the subgroup receiving alternative therapy. Two patients received anidulafungin therapy for 9 and 18 days, respectively; one patient received caspofungin therapy for 13 days; and one patient received 10 days of micafungin therapy. The remaining 27 patients received voriconazole each for a total dose ranging from 1000 mg (plus 2 days of liposomal amphotericin B) to 18,300 mg, with a mean value of 6016 mg (standard deviation, 4990 mg). In comparison to 4 deaths (44.4%) of the 9 patients without adequate treatment, 16 of 31 (51.6%) of the treatment group patients died, or 14 of 27 (51.9%) voriconazole treatment subgroup died, and thus the difference of mortality rate relevant to antifungal therapy did not reach statistical significance (44.4% vs. 51.6%, *p* = 0.705; or 44.4% vs. 51.9%, *p* = 0.700, respectively). The mortality rate of patients without any antifungal therapy (1/6, 16.7%) was apparently lower than that of those in the treatment group (51.6%) or the voriconazole treatment subgroup (51.9%,), but the difference did not reach statistical significance (*p* = 0.116 and *p* = 0.117, respectively) by using the Yates correction.

### 3.5. Risk Factors for Mortality of Group 1 Patients (Flu with IPA)

The overall mortality of group 1 patients was 50%. Univariate analysis revealed risk factors for mortality of group 1 patients (Table 3), including Charlson comorbidity index (*p* = 010), APACHE II score (*p* = 0.001), and severe ARDS (*p* = 0.038), the odds ratios for which were all statistically significant when using multivariable regression analysis (Table 4).

### 3.6. Risk Factors for Mortality of Group 1 and Group 2 Patients (Severe Flu with and without IPA)

To further clarify whether IPA significantly contributes to the mortality of confirmed severe influenza patients in combined group 1 and group 2, a logistic regression model was performed. IPA contributed an odds ratio of 7.33 (*p* < 0.001) for mortality in comparison to influenza without IPA using univariate analysis and an odds ratio of 13.78 (*p* < 0.001) for mortality by using multivariate analysis (Table 5). The other potential risk factors for mortality revealed statistical significance in APACHE II score (*p* = 0.001), SOFA score (*p* = 0.029), severe ARDS (*p* < 0.001), and bacterial coinfections within 2 days of admission (*p* = 0.027) using univariate analysis, whereas only IPA, Charlson comorbidity index, APACHE II score, and severe ARDS were all independent risk factors for mortality of severe influenza patients by using multivariate analysis (Table 5).

## 4. Discussion

We recently reported an increasing trend of IPA over five years in southern Taiwan, which was epidemiologically correlated with the trends of influenza, especially influenza A (H1N1) [21]. In this study, we further investigated the impacts of IPA on the clinical outcomes of patients with severe influenza, which might help physicians to drive appropriate strategies in clinical pathways. There was a potential for delayed diagnosis of IPA in clinical practice, as influenza was diagnosed on average 2 days after admission in the studied institutes, and IPA was diagnosed after a mean of 7.5 days post diagnosing influenza. Delayed treatment of influenza-associated IPA might contribute to a high mortality [22]. Nonetheless, some IPA occurrences might probably be late-onset diseases; that is, secondary infections but not real “coinfections” of IPA at early onset of influenza infections. In fact, we did intend to differentiate between IPA secondary infection or “coinfection” with influenza in this study. We only defined bacterial “coinfection” within 2 days of diagnosing influenza infection. In addition to influenza and IPA, bacterial coinfection was not uncommon in this study. The rate of bacterial coinfection was found to range from 38.1% to 45%; furthermore, 18 (45%) patients had multiple coinfections of influenza, IPA, and bacteria. Although significant in univariate analysis, bacterial coinfection was not significantly associated with mortality in severe influenza patients using multivariate analysis.

We determined several significant findings. Firstly, coinfection with IPA could adversely affect the outcomes of severe influenza patients, and these adverse impacts included prolonged ICU stay (>21 days) and hospital stay as well as increased mortality and medical cost. Most importantly, we found that influenza patients without testing for GM (group 3) had similar outcomes to influenza patients with and without IPA coinfection by using survival analysis, which might suggest the heterogenous characteristics and the possible underdiagnosis of IPA in these patients (group 3). Nonetheless, the overall prevalence of IPA in severely affected influenza patients would be at least 19.9% (40/201), similar to previous reports of the prevalence up to 16–23% globally [9,12,14,15]. Thus, clinicians should maintain vigilance for the possible occurrence of coinfected IPA among critically ill patients with influenza.

Secondly, in line with previous studies investigating the coinfection between IPA and influenza among critically ill patients, the morbidity and mortality of this population were high [7,9,11,16,23]. Septic shock, acute kidney injury, and acute respiratory failure requiring mechanical ventilation were common complications, and the mortality rate was as high as 50% in this study. The influenza without testing for GM (group 3) patients had significantly lower SOFA scores and less requirement of mechanical ventilation than the other two groups of influenza patients, which might reasonably hinder the motivation of physicians to order GM testing.

Thirdly, six patients in group 1 were not treated with any antifungal agents, probably due to relatively low GM level, moderate but not severe ARDS, no required ventilator support, rapid resolution of pneumonia, or rapid weaning from ventilator support, so that attending physicians might decide that there is no need to initiate antifungal therapy. Although five of these patients survived without antifungal therapy, nonetheless, they might suffer from substantial morbidity, such as prolonged hospital stay or prolonged course of resolution of pulmonary infiltrates even after discharge. Therefore, continued IPA infection in a few influenza patients might not need antifungal therapy as they could have recovered from influenza-associated transient immune impairment, but it does not necessarily mean that the IPA diagnosis was false positive. Therefore, we did not manipulate these five patients into the group 2 category. Since the mortality rate of the non-treatment group was not significantly different from that of the treatment group or voriconazole subgroup, it would be difficult to suggest which influenza patients who fulfil the IPA diagnosis might not need antifungal treatment. Furthermore, we identified three significant risk factors for the mortality of severe influenza patients with IPA, including patients’ underlying comorbidity (Charlson comorbidity index), initial presentation of disease severity (APACHE II score), and disease complication with severe ARDS. Meanwhile, IPA was an independent risk factor for mortality in severe influenza patients in addition to the above-mentioned risk factors. Therefore, we suggest prompt antifungal therapy according to the presence of risk factors for mortality.

Finally, the proportions of solid cancer, severe ARDS, CMV DNAemia, corticosteroid use, and mortality in group 1 patients with coinfected IPA and influenza were significantly higher than that in the other two groups of influenza patients. Nonetheless, only solid cancer and prolonged corticosteroid use in ICU of >5 days had a statistically significant odds ratio of IPA occurrence in severe influenza patients, highlighting the need for GM testing in severely ill influenza patients, especially with host factors of solid cancer and corticosteroid use. Meanwhile, our data might indicate severe ARDS and CMV infection as disease consequences of IPA but not as initiating events to the IPA in a substantial number of cases, so that confounded the odds ratio of severe ARDS and CMV to play a significant role of risk for IPA occurrence. However, it is worth noting that Kuo et al., reported CMV viremia as an independent risk factor of IPA in critically ill patients, while an additive synergistic effect for IPA risk was found between CMV viremia and influenza [24].

There are several limitations in this study. Firstly, a positive GM index in serum (>0.5) or in BAL (≥1.0) has been proposed for influenza-associated pulmonary aspergillosis based on the cost-effective evaluation [10]. However, our GM cutoff index of ≥0.5 in serum or ≥0.7 in BAL in real-world practice has offered statistical significance to differentiate clinical outcome between influenza patients with and without IPA. Secondly, five patients in group 1 survived without any antifungal therapy, which might hint false-positive GM testing and that they should have been enrolled into group 2. However, our potential wrong grouping could not eventually change the trend of significantly higher mortality of group 1 than group 2 patients. Thirdly, as there were a limited number of cases with and without antifungal therapy, we cannot confirm the impact of antifungal therapy on the clinical outcome of IPA in influenza patients, which mandates further prospective study based on standardized and programmatic diagnostic and therapeutic protocol.

In conclusion, IPA imposed significant adverse impacts on severely ill influenza patients, leading to substantially high mortality. Accordingly, physicians should pay more attention to make an earlier initiation of IPA diagnostic processes for critically ill patients with influenza, particularly with the host risk factors for acquiring aspergillosis, such as solid cancer and prolonged steroid use in ICU. Prompt initiation of antifungal therapy subsequently is mandatory based on risk factors for mortality.

## Figures and Tables

**Table 1:** Clinical characteristics of ICU patients with severe influenza in comparison among those with aspergillosis (group 1), negative galactomannan assay (group 2), and non-testing for galactomannan (group 3).

Variables	Group 1 (*n* = 40)	Group 2 (*n* = 50)	Group 3 (*n* = 111)	*p* Value
Hospital (CMMC), no. (%)	31 (77.5)	37 (74)	53 (47.7) *	**<0.001**
Female, no. (%)	16 (40)	20 (40)	43 (38.7)	0.984
Age (mean ± SD)	60 ± 14 *	66 ± 13	70 ± 14	**<0.001**
BMI (mean ± SD)	24.5 ± 4.8	25 ± 4.7	23.9 ± 6.1	0.496
Underlying diseases, no. (%)				
Diabetes mellitus	19 (47.5)	20 (40)	42 (37.8)	0.565
Chronic obstructive pulmonary disease	2 (5)	3 (6)	17 (15.3)	0.087
Solid cancer	9 (22.5) *	2 (4)	10 (9)	**0.013**
ESRD with maintenance dialysis	3 (7.5)	6 (12)	11 (9.9)	0.815 F
Liver cirrhosis	3 (7.5)	5 (10)	4 (3.6)	0.233 F
Hematological malignance	1 (2.5)	0	2 (1.8)	0.771 F
Solid organ transplant recipient	2 (5)	1 (2)	0 (0)	0.051 F
HIV infection	0	0	0	-
Autoimmune disease	0	0	0	-
Charlson comorbidity index (mean ± SD)	4.4 ± 2.5	5.2 ± 2.7	5.2 ± 2.2	0.161
Steroid (prednisolone or equivalent), no. (%)				
Long-term use, >0.3 mg/kg/day, >3weeks	0	0	0	
Within 3 weeks, >5 mg/day, >7 days	1 (2.5)	2 (4)	1 (0.9)	0.308 F
In ICU, >5 mg/day, >5 days	31 (77.5) *	22 (44)	48 (43.2)	**0.001**
Daily dose (mg)	30.6 ± 13.3	27 ± 17.2	25.1 ± 19	0.230
Total dose (mg)	629.5 ± 507.3	424.4 ± 430.5	170.7 ± 199.1 *	**<0.001 W**
Duration (day)	19.9 ± 15.8	14.7 ± 16	5.5 ± 6.8 *	**<0.001 W**
Temperature (°C) on admission (mean ± SD)	37 ± 0.7	37.1 ± 0.8	37 ± 0.7	0.746
Fever (≥38 °C) on admission, no. (%)	3 (7.5)	6 (12)	13 (11.7)	0.826
Severity status, no. (%)				
APACHE II score (mean ± SD)	21.1 ± 10.4	18.0 ± 8.5	18.1 ± 8.2	0.236 W
SOFA score (mean ± SD)	7.7 ± 4.4 *	6.7 ± 4.2 ^a^	5.5 ± 5 ^a^	**0.030**
Ventilator use	25 (62.5)	33 (66)	41 (36.9) *	**0.001**
On ECMO	1 (2.5)	0	1 (0.9)	0.420 F
Complications, no. (%)				
Septic shock	39 (97.5)	48 (96)	106 (95.5)	1.000
Bloodstream infection	7 (17.5)	4 (8)	8 (7.2)	0.158
Acute kidney injury (Creatinine > 2 mg/dL)	15 (37.5)	10 (23.3)	32 (30.8)	0.369
Acute jaundice (Total bilirubin > 2 mg/dL)	4 (16.7)	2 (6.9)	3 (5.9)	0.339 F
Platelet count <100,000/μL	7 (17.9)	6 (12)	17 (15.3)	0.731
Blood PCR for *Cytomegalovirus* DNA	7 (17.5) *	4 (8) ^a^	1 (0.9) ^a^	**0.006**
Chest X-ray finding				
Normal	None	None	None	-
Peribronchial infiltrations	2 (5)	2 (4)	14 (45.9)	0.129
Bilateral lung patch infiltrates	7 (17.5)	12 (24)	51 (45.9) *	**<0.001**
Multiple patches with necrotizing processes	10 (25)	17 (34)	29 (26.1)	0.531
Diffuse ground-glass appearance	6 (15)	9 (18)	9 (8.1)	0.161
Extensive consolidation on one lung	1 (2.5)	2 (4)	7 (6.3)	0.596
Extensive consolidation on bilateral lungs	11 (27.5) *	6 (12) ^a^	1 (0.9) ^a^	**<0.001**
Diffuse air-space infiltration pattern	3 (7.5)	2 (4)	0 (0)	-
ARDS, no. (%)	33 (82.5) *	32 (66.7)	58 (63)	**0.003**
Mild	5 (12.5)	11 (22.9)	25 (27.2)	0.382
Moderate	16 (40)	12 (25)	26 (28.3)	0.111
Severe	12 (30) *	9 (18.8)	7 (7.6)	**<0.001**
PaO2/FiO2 ratio (mean ± SD)	210 ± 185 *	259 ± 231	310 ± 217	**0.034**
Bacterial coinfections (within 2 days), no. (%)	18 (45)	20 (41.7)	37 (38.1)	0.746
Influenza assay				
RIDT (negative result)	14 (35) *	11 (22)	18 (16.2)	**0.045**
Influenza A (H1N1)	15 (37.5)	19 (38)	26 (23.4)	0.087
Influenza A (H3N2)	3 (7.5) *	19 (38) ^a^	71 (64) ^a^	**<0.001**
Influenza A (negative for H1N1 and H3N2)	15 (37.5) *	7 (14)	6 (5.4)	**<0.001**
Influenza B	7 (17.5)	5 (10)	8 (7.2)	0.176
Inflammatory markers				
White blood cell count/μL (mean ± SD) × 1000	11.1 ± 6.2	10.7 ± 5.1	10.9 ± 7.1	0.944
Lymphopenia (<1000/μL), no. (%)	33 (82.5) *	33 (66)	67 (64.4)	**0.040**
C-reactive protein (mean ± SD)	135.3 ± 129	105.6 ± 80.8	42 ± 54	0.051
Procalcitonin (mean ± SD)	22.8 ± 45.5	25 ± 51.4	18.5 ± 37.1	0.788
Platelet count/μL (mean ± SD) × 1000	175.4 ± 79.5	182.5 ± 74.5	172.4 ± 82.5	0.759
Antiviral therapy				
Oseltamivir, no. (%)	37 (92.5)	45 (90)	99 (89.2)	0.903
Peramivir, no. (%)	6 (15)	7 (14)	22 (19.8)	0.602
Antifungal therapy				
Voriconazole, no. (%)	27 (67.5) *	1 (2)	1 (0.9)	**<0.001**
Caspofungin, no. (%)	8 (20)	0 (0)	0 (0)	-
Liposomal amphotericin B, no. (%)	3 (7.5)	0 (0)	0 (0)	-
Anidulafungin, no. (%)	3 (7.5)	0 (0)	0	-
Clinical outcome				
ICU stay >21 days, no. (%)	20 (50) *	7 (14)	8 (7.2)	**<0.001**
Hospitalization day (mean ± SD)	37 ± 21.4 *	26.7 ± 16.1	16.1 ± 12.6	**<0.001 W**
Overall death, no. (%)	20 (50) *	6 (12)	18 (16.2)	**<0.001**
Disease economic burden (NTD)				
Hospital cost (mean ± SD)	339,133 ± 245,959 *	112,391 ± 81,738	64,620 ± 99,841	**<0.001 W**
Drug fee (mean ± SD)	256,782 ± 199,789 *	59,285 ± 57,589	28,793 ± 64,565	**<0.001 W**
Laboratory fee (mean ± SD)	82,350 ± 103,220 ^a^	53,105 ± 36,953	35,826 ± 60,280 ^a^	**0.010 W**

Note: ICU, intensive care unit; CMMC, Chi Mei Medical Center; BMI, body mass index; F, Fisher’s exact test; ESRD, end-stage renal disease; HIV, human immunodeficiency virus; W, Welch’s ANOVA test; APACHE, Acute Physiology and Chronic Health Evaluation; SOFA, Sequential Organ Failure Assessment; ECMO, extracorporeal membrane oxygenation; PCR, polymerase chain reaction; ARDS, acute respiratory distress syndrome; RITD, rapid influenza diagnostic test; NTD, New Taiwan Dollar. Superscript: * Significant factor when further compared with the other variants; ^a^ Significantly different when variants marked “a” in superscript are compared. Bold indicates *p* < 0.05.

**Table 2:** The clinical characteristics of IPA in severe influenza patients who were not given any antifungal therapy (*n* = 6) or were not given it in time to treat, such that patients died within one day of therapy (*n* = 3), that could be arbitrarily regarded as inadequate antifungal therapy. Four patients received an echinocandin for alternative therapy.

Patient	Age. Sex	Ventilator Use	ARDS	P/F Ratio	Day of IPA Dx Post Influenza (d)	Total Dose (mg)	Outcome	Hospital Stay (d)	Serum GM Index	BAL GM Index
No therapy										
Patient 7	38 F	nil	moderate	165.6	23	0	survived	68	0.60	NA
Patient 10	57 M	nil	moderate	155.6	9	0	survived	39	0.50	NA
Patient 17	58 F	nil	moderate	124	6	0	survived	17	0.78	NA
Patient 20	61 F	yes	moderate	162	10	0	died	20	0.65	NA
Patient 32	64 F	nil	moderate	141.6	2	0	survived	20	3.38	NA
Patient 38	48 M	yes	nil	694	1	0	survived	9	1.24	0.19
Inadequate therapy										
Patient 3	45 F	yes	moderate	184	7	C 70	died	4	0.56	NA
Patient 25	59 M	yes	severe	86.13	64	V 900	died	71	1.93	NA
Patient 30	59 M	nil	mild	259.7	2	V 300	died	4	3.35	NA
Alternative therapy										
Patient 27	56 F	nil	mild	214.3	33	A 500	died	50	1.99	NA
Patient 33	60 M	yes	moderate	172.67	1	M 1000	survived	31	0.69	NA
Patient 36	60 M	yes	nil	382.67	14	C 670	died	39	13.63	NA
Patient 40	36 F	yes	nil	504	6	A 3600	survived	25	0.14	8.19

Note. ARDS, acute respiratory distress syndrome; P/F ratio, PaO2/FiO2 ratio; IPA, invasive pulmonary aspergillosis; GM, galactomannan; BAL, bronchoalveolar lavage; C, caspofungin; V, voriconazole; A, anidulafungin; M, micafungin; NA, not available.

**Table 3:** Risk factors for mortality in ICU patients with coinfection of influenza and aspergillosis (group 1) using univariate analysis.

Variables	Group1 (*n* = 40)	Survival (*n* = 20)	Death (*n* = 20)	*p* Value
Female, no. (%)	16 (40)	9 (56.3)	7 (43.7)	0.748
Age (mean ± SD)	60 ± 14	55.9 ± 13.6	64.5 ± 13.3	0.050
BMI (mean ± SD)	24.5 ± 4.8	25.0 ± 5.2	24.1 ± 4.3	0.560
Underlying diseases, no. (%)				
Diabetes mellitus	19 (47.5)	7 (36.8)	12 (63.2)	0.205
Solid cancer	9 (22.5)	3 (33.3)	6 (66.6)	0.451 F
ESRD with maintenance dialysis	3 (7.5)	2 (66.7)	1 (33.3)	1.000 F
Liver cirrhosis	3 (7.5)	0 (0)	3 (100)	0.231 F
Charlson comorbidity index (mean ± SD)	4.4 ± 2.5	3.4 ± 2.4	5.4 ± 2.3	0.010
Steroid (prednisolone or equivalent), no. (%)				
Daily dose (mg)	30.6 ± 13.3	29.2 ± 16.9	32.0 ± 8.5	0.510
Total dose (mg)	629.5 ± 507.3	544.4 ± 511.5	714.5 ± 501.3	0.295
Duration (day)	19.9 ± 15.8	16.9 ± 15.4	23.0 ± 16.1	0.228
Inflammatory markers				
Lymphopenia (<1000/μL), no. (%)	33 (82.5)	17 (51.5)	16 (48.5)	1.000 F
C-reactive protein (mean ± SD)	135.3 ± 129	168.3 ± 165.5	105.8 ± 78.0	0.149
Procalcitonin (mean ± SD)	22.8 ± 45.5	21.6 ± 49.8	23.9 ± 42.5	0.886
Platelet count/μL (mean ± SD) × 1000	175.4 ± 79.5	185.3 ± 61.5	166.0 ± 94.1	0.451
Severity status, no. (%)				
APACHE II score (mean ± SD)	21.1 ± 10.4	15. 8± 8.4	26.4 ± 9.6	0.001
SOFA score (mean ± SD)	7.7 ± 4.4	6.9 ± 4.1	8.6 ± 4.6	0.216
Ventilator use	25 (62.5)	12 (48)	13 (52)	1.000
Septic shock	39 (97.5)	20 (51.3)	19 (48.7)	1.000 F
ARDS, no. (%)	33 (82.5)	16 (48.5)	17 (51.5)	1.000 F
Severe ARDS, no. (%)	12 (30)	3 (25)	9 (75)	0.038
PaO2/FiO2 ratio (mean ± SD)	210 ± 185	233.3 ± 156.2	197.2 ± 212.4	0.661
Bacterial coinfections (within 2 days), no. (%)	18 (45)	7 (38.9)	11 (61.1)	0.341
Blood PCR for *Cytomegalovirus* DNA	7 (17.5)	3 (42.9)	4 (57.1)	1.000 F
Influenza assay				0.588
Influenza A (H1N1)	15 (37.5)	8 (53.3)	7 (46.7)	
Influenza A (H3N2)	3 (7.5)	1 (33.3)	2 (66.7)	
Influenza A (negative for H1N1 and H3N2)	15 (37.5)	9 (60)	6 (40)	
Influenza B	7 (17.5)	2 (28.6)	5 (71.4)	
Diagnosis criteria for aspergillosis				
BAL galactomannan level (index)	2.4 ± 3.4	1.6 ± 3.2	4.9 ± 3.6	0.262
Serum galactomannan level (index)	1.5 ± 2.3	1.1 ± 1.3	2.0 ± 3.2	0.272
Antiviral therapy				
Oseltamivir, no. (%)	37 (92.5)	18 (48.6)	19 (51.4)	1.000 F
Peramivir, no. (%)	6 (15)	4 (66.7)	2 (33.3)	0.661 F
Antifungal therapy				
Voriconazole, no. (%)	27 (67.5)	11 (42.3)	15 (57.7)	0.320
Caspofungin, no. (%)	8 (20)	2 (25)	6 (75)	0.235 F

Note: ICU, intensive care unit; BMI, body mass index; F, Fisher’s exact test; ESRD, end-stage renal disease; APACHE, Acute Physiology and Chronic Health Evaluation; SOFA, Sequential Organ Failure Assessment; PCR, polymerase chain reaction; ARDS, acute respiratory distress syndrome; BAL, bronchoalveolar lavage.

**Table 4:** Risk factors for mortality in ICU patients with coinfection of influenza and aspergillosis (group 1) using multivariate regression analysis.

Variable	Crude Odds Ratio (95% CI)	*p* Value	Adjusted Odds Ratio (95% CI)	*p* Value
ARDS				
Non-severe	1.00		1.00	
Severe	4.636 (1.023–21.004)	0.047	24.774 (1.913–320.859)	0.014
Charlson comorbidity index	1.467 (1.064–2.023)	0.019	1.569 (1.058–2.327)	0.025
APACHE II score	1.128 (1.042–1.221)	0.003	1.150 (1.038–1.275)	0.008

**Table 5:** Risk factors for in-hospital mortality (*n* = 26) of severe influenza patients with and without aspergillosis (groups 1 and 2, *n* = 90) determined by using logistic regression model.

Variables	Univariate Analysis			Multivariate Analysis		
	Odds Ratio	95% CI	*p* Value	Odds Ratio	95% CI	*p* Value
Clinical group						
Group 1: Flu with IPA	7.33	2.56–21.05	<0.001	13.78	3.19–59.5	<0.001
Group 2: Flu without IPA	1.00			1.00		
Solid cancer	3.54	0.97–12.8	0.055			
Charlson comorbidity index	1.16	0.98–1.39	0.094	1.41	1.07–1.86	0.015
APACHE II score	1.10	1.04–1.17	0.001	1.10	1.02–1.19	0.010
SOFA score (mean ± SD)	1.13	1.01–1.26	0.029			
ARDS						
Severe degree	7.00	2.41–20.36	<0.001	18.45	3.55–95.83	0.001
Non-severe	1.00			1.00		
Bacterial coinfection	2.91	1.13–7.49	0.027			

Note. Flu, influenza; APACHE, Acute Physiology and Chronic Health Evaluation; ARDS, acute respiratory distress syndrome.

## Data Availability

The data that support the findings of this study are available on request from the corresponding author.
